# A novel optimal identification of various solar PV cell parameters by using MRDT controller

**DOI:** 10.1038/s41598-024-61359-x

**Published:** 2024-05-07

**Authors:** Sunkara Sunil Kumar, K. Balakrishna

**Affiliations:** Vignan’s Foundation for Science Technology and Research, Vadlamudi, India

**Keywords:** Accuracy, Convergence rate, Dichotomy, RMSE, Single diode cell, 2-Didoes based solar cell, 3-Diodes based solar cell, Plus modified Rao technique, Engineering, Electrical and electronic engineering

## Abstract

At present, Renewable Energy Sources (RES) utilization keeps on increasing because of their merits are more availability in the atmosphere, easy energy harvesting, less maintenance expenses, plus more reliability. Here, the solar power generation systems are utilized for supplying the energy to the local consumers. The accurate, and efficient solar power supply to the customers is a very important factor to meet the peak load demand. The accurate power generation of the sunlight system completely depends on its accurate parameters extraction. In this work, a Modified Rao-based Dichotomy Technique (MRAODT) is introduced to identify the actual parameters of the different PV cells which are PWP 201 polycrystalline, plus RTC France. The proposed MRAODT method is compared with the other existing algorithms which are the teaching and learning algorithm, African vultures, plus tuna intelligence algorithm. Finally, from the simulation results, the MRAODT gives superior performance when associated with the other controllers in terms of parameters extraction time, accuracy in the PV cells parameters identification, plus convergence time of the algorithm.

## Introduction

From the literature review, the usage of non-renewable energy sources is decreasing drastically because their drawbacks are more atmospheric pollution, extreme greenhouse gas emissions, more catchment area needed for developing the power plants, plus the input fuel transportation is also very high^1^. So, the researchers working on the development of renewable energy systems. The features of renewable power networks are low levels of atmospheric pollution, easy installation, fewer human sources required for maintenance, plus high robustness. Also, renewable energy is available in the environment very excessively. So, the input fuel supply cost is zero for RESs. The major renewable power systems are geothermal, wind, hydropower, tidal, plus sunlight networks. The hydropower stations work based on the water head. High water head gives more kinetic energy thereby extracting the hydro power is also more. Sometimes, the storage of water is absent then the water head level is reduced^2^. As a result, the power utilization from the hydro system is limited.

So, the wind power stations are applied to the smart grid systems to enhance the functioning efficiency of the overall distribution power system. Here, the wind plants collect the wind kinetic energy by using the wind turbines. Wind flow over the blades generates the lift to rotate the wind blades^3^. The wind blades are directly integrated with the rotor shaft for functioning the electric generator. The generator transfers the wind blades' rotational energy into the useful power supply. The features of wind systems are less carbon dioxide emission, freely available on the earth, easy operation, very simple equipment, plus less effect on atmospheric conditions^4^. However, this wind system should be located near remote locations because of its high noise creation. Also, this energy source is unpredictable plus highly dangerous to wildlife. So, the geothermal power supply strategy is used in automotive systems for continuous power supply to the battery charging application^5^. In this geothermal network, the water content is transferred into the steam. The evaporated steam is directly sent to the steam turbine chamber to run the electrical generator. The merits of geothermal networks are very silent, always available, and have less impact on human beings, plus good energy density. However, it is less stable on the earth, plus more maintenance costs^6^.

**Figure 1:**
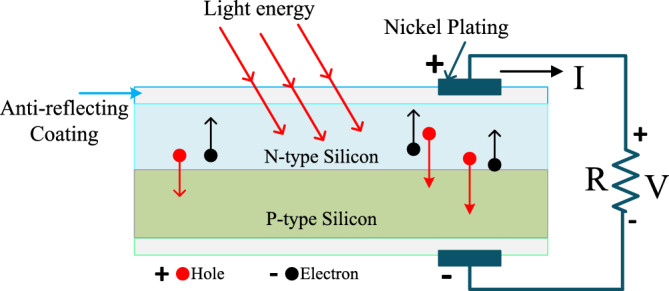
Schematic representation of the sunlight system^10^.

The fuel stack-based electrical energy-generated systems are utilized in the article^7^ for stabilizing the voltage of the automotive systems. From the literature study, the fuel stacks are suitable for stationary power generation, and transportation applications. The merits of fuel stack systems are more energy efficient, have zero emissions, are highly robust, more scalable, plus low operating costs^8^. However, this system's implementation cost is higher. So, the solar systems are integrated with the already existing power supply networks to meet the peak load consumer demand. A solar system is the most powerful and useful energy source for rural area people. The working structure of the sunlight system is provided in Fig. 1. Here, there are various categories of sunlight systems have existed in the literature which are thin film, silicon-based monocrystalline, copper indium gallium selenide, Cadmium telluride, plus Polycrystalline solar panels^9^. The solar networks are developed by utilizing the 1-diode, 2-diodes, plus 3-diodes-based solar cells. Here, the major issue of the sunlight system is nonlinear performance. As a result, the extraction of voltage from the sunlight system is quite a difficult task. In addition, the accurate parameter identification of solar cells is difficult^10^. So, there are different categories of optimization methodologies are exist in the literature to obtain the accurate parameters of the solar cell thereby enhancing the efficiency of the overall system.

**Figure 2:**
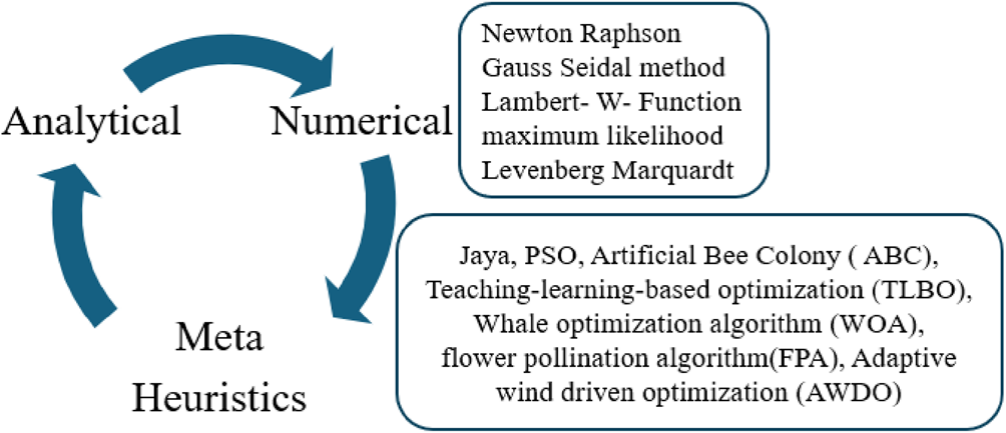
Various algorithms for the extraction of solar cell parameters^12^.

In Ref.^11^, the authors explained that the total number of parameters required for the implementation of one diode solar cell is five which are named solar photocurrent (I_ph_), ideality factor of the concern diode (ʎ), the saturation current of the circuit (I_0_), shunt resistive element (R_sh_), plus series placed resistive element (R_s_). Here, the sunlight system performance and identification of suitable solar cell parameters are obtained by using its nonlinear V–I and P–V curves. The suitable parameters of the solar cell on the V–I curve are determined by applying various natural inspiration-based optimization algorithms^12^. The classification of swarm intelligence-associated algorithms and their application on solar parameters extraction is illustrated in Fig. 2. From Fig. 2, most of the research scholars worked out on the one diode-based sunlight system for enhancing its functioning efficiency by evaluating the accurate short circuit current, and series resistance of the circuit. The features of the 1-diode circuit model are easy implementation, few factors required for the development, less mathematical computation is needed, plus the low cost of installation^13^.

From the literature review, the solar cell parameters are determined by selecting the numerical, soft computing, and metaheuristic algorithms. Among all of the parameter extraction methodologies, metaheuristic techniques are the predominant methods when associated with the other methods because their merits are more accurate in the parameter selection of the solar cells, easy way to enhance the efficiency of the sunlight power system, plus more reliability. In Ref.^14^, the authors estimated the 2-diode model PV circuit variables which are named reverse saturation current of the diode one (I_01_), saturation current of the diode (I_02_), ideality factors of the diode’s variables (ʎ_1_ and ʎ_2_), shunt resistive element (R_sh_), photocurrent (I_ph_), plus series placed resistive element (R_s_).

In Ref.^15^, the authors studied all over three solar cell technologies in terms of parameter extraction, and operating efficiency. From the simulative study, the authors decided that the single plus double diode methodologies do not provide accurate results when associated with the triple diode cell technology. In this triple-diode circuit, the leakage currents of the PV cell are also considered for the designing of the three-diode-based sunlight system, and its related implementation data is collected from the datasheet given by the manufacturers at Standard Test Condition (STC). The major variables utilized to extract any solar cell parameters are the open circuit voltage point on the V–I curve, peak power, peak current available from the sunlight system, plus short-circuited current point^16^. The analytical methods give low-level accurate PV system parameters when associated with the metaheuristic, plus arithmetical methods. Suppose, any changes in the system conditions then the evaluation of the parameters may not be proper and the solar cells' mathematical equations are developed by approximating the various sunlight conditions.

In Ref.^17^, the authors utilized curve fitting, Particle Swarm Intelligence (PSI), Jaya, Flower Pollicization (FP), High Convergence PSO, and Simulated Annealing Algorithms for the identification of solar cell parameters with variable step sizes. However, these methods have the drawback of high complexity in development. So, the stochastic methodologies are involved with the numerical techniques for enhancing the functioning efficiency of the sunlight systems. Here, the hybrid methods evaluate the global solution instead of local optima, and it gives a wide range of solutions for the parameter extraction of the sunlight system. The Newton-Rapson concept is integrated with the chaotic methods in Ref.^18^ to reduce the overall controller iteration value. As a result, the system works effectively with an optimal number of iterations.

In the article^19^, the authors discussed the Levenberg, plus Wind Driven Optimization (WDO) methodologies for reducing the fluctuations in the system performance. The disadvantage of WDO-dependent methodologies is slow convergence rate for identifying the global optima. So, the Elephant Herd Algorithm (EHA) is proposed in Ref.^20^ for verifying the parameters extraction of the sunlight system by using the Genetic Optimization Grey Wolf method. However, the limitations of the above methods are low accuracy levels, more fluctuations in the system behavior, plus less suitability for the three diode-based sunlight systems. Here, the modified Rao methodology is utilized for the suitable parameters.

## Mathematical development of solar PV cells

**Figure 3:**
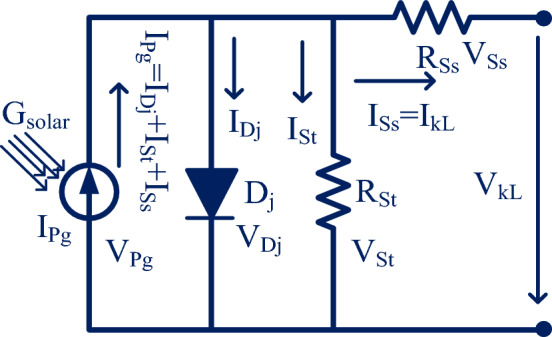
1-diode sunlight PV circuit with irradiations.

From the solar cell manufacturers, the solar system is developed by placing the photocurrent in parallel with the diode. Here, the photocurrent is obtained by combining the two silicon materials which are P-type, plus N-type materials. Whenever the sunlight hits the power semiconductor materials then the freely running electrons in the materials observe the sunlight insolations for moving from one side layer of the P-N diode to another layer^21^. Here, the diode works to eliminate the reverse leakage currents of the sunlight network which is discussed in Fig. 2. From Fig. 3, the diode (D_j_) improves the sunlight system efficiency by limiting the over-current flow in the network. The parameters I_Dj_, I_Ph_, I_St_, I_Ss_, plus I_Lk_ are the diode current, photocurrent, shunt current flow, series current flow, plus load current flow. The elements R_St_, plus R_Ss_ are the shunt connected and series connected resistances.

**Figure 4:**
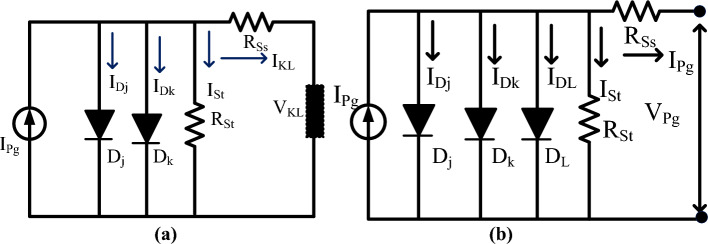
Schematic representation of (**a**) 2-diode, plus (**b**) 3-diode PV circuits.

The available photocurrent from the circuit is discussed in Eq. (1). Suppose, there are multiple cells considered in the sunlight system then Eq. (3) is applied to determine the sunlight current. Here, Eq. (8) is considered for evaluating the sunlight current by neglecting the shunt resistance of the PV circuit. Finally, the PV circuit involves all the resistive elements then the available PV module current is obtained by selecting Eq. (12), plus Eq. (13). Similar to the 1-diode model, the 2-diode model, plus 3-diode model sunlight systems PV circuits are illustrated in Fig. 4a, plus b. From Fig. 4a, the extra diode is placed in parallel to the photocurrent for working the solar module with high accuracy at very low irradiation conditions. The addition of a diode with the existing 1-diode circuit PV system creates complexity in the system. Also, its installation, and manufacturing costs are increased. So, there are various advanced PV technologies available in the present market for optimizing the cost by identifying accurate solar system parameters.

1 $${{\text{I}}}_{{\text{Lk}}}={{\text{I}}}_{{\text{Pg}}}-{{\text{I}}}_{{\text{revc}}}\left({{\text{e}}}^{\left(\frac{{{\text{V}}}_{{\text{Pg}}}*{\text{q}}}{\uplambda *{\text{K}}*{\text{T}}}\right)}-1\right)$$

2 $${{\text{I}}}_{{\text{Pg}}}=\left({{\text{I}}}_{{\text{PgSTC}}}+{{\text{K}}}_{{\text{l}}} ({({\text{T}}-{\text{T}})}_{0}\right)*\frac{{{\text{G}}}_{{\text{irra}}}}{{{\text{G}}}_{{\text{irra}}}{\text{STC}}}$$

3 $${{\text{I}}}_{{\text{Lk}}}={{\text{I}}}_{{\text{Pg}}}-{{\text{I}}}_{{\text{revc}}}\left({{\text{e}}}^{\left(\frac{{{\text{V}}}_{{\text{Pg}}}*{\text{q}}}{\uplambda *{\text{K}}*{\text{T}}*{{\text{N}}}_{{\text{S}}}}\right)}-1\right)$$

4 $${{\text{I}}}_{{\text{sc}}\_{\text{solar}}}={{\text{I}}}_{{\text{LK}}}={{\text{I}}}_{{\text{Pg}}}$$

5 $${{\text{I}}}_{{\text{sc}}\_{\text{Solar}}}={{\text{I}}}_{{\text{Pg}}}={{\text{I}}}_{{\text{revc}}}\left({{\text{e}}}^{\left(\frac{{{\text{V}}}_{{\text{Pg}}}*{\text{q}}}{\uplambda *{\text{K}}*{\text{T}}*{{\text{N}}}_{{\text{S}}}}\right)}-1\right)$$

6 $${{\text{V}}}_{{\text{oc}}\_{\text{Solar}}}=\frac{\uplambda *{\text{K}}*{\text{T}}*{{\text{N}}}_{{\text{S}}}}{{\text{q}}}{\text{log}}\left(\frac{{{\text{I}}}_{{\text{Pg}}}}{{{\text{I}}}_{{\text{revc}}}}+1\right)$$

7 $${{\text{P}}}_{{\text{Pg}}}={{\text{I}}}_{{\text{Pg}}}*{{\text{V}}}_{{\text{Pg}}}=\left({{\text{I}}}_{{\text{Pg}}}-{{\text{I}}}_{{\text{revc}}}\left({{\text{e}}}^{\left(\frac{{{\text{V}}}_{{\text{Pg}}}*{\text{q}}}{\uplambda *{\text{K}}*{\text{T}}*{{\text{N}}}_{{\text{S}}}}\right)}-1\right)*{{\text{V}}}_{{\text{LK}}}\right)$$

8 $${{\text{I}}}_{{\text{LK}}}={{\text{I}}}_{{\text{Pg}}}-\left({{\text{I}}}_{{\text{revc}}}\left({{\text{e}}}^{\left(\frac{{{\text{V}}}_{{\text{Pg}}}*{\text{q}}+{{\text{I}}}_{{\text{Pg}}*}{{\text{N}}}_{{\text{s}}}*{{\text{R}}}_{{\text{ss}}}}{\uplambda *{\text{K}}*{\text{T}}*{{\text{N}}}_{{\text{S}}}}\right)}-1\right)-1\right)$$

9 $${{\text{V}}}_{{\text{oc}}\_{\text{solar}}}=\frac{{{\text{N}}}_{{\text{s}}}\uplambda *{\text{K}}*{\text{T}}}{{\text{q}}}{\text{log}}\left(\frac{{{\text{I}}}_{{\text{Pg}}}}{{{\text{I}}}_{{\text{revc}}}}+1\right)$$

10 $${{\text{I}}}_{{\text{sc}}-{\text{solar}}}={{\text{I}}}_{{\text{Pg}}}-{{\text{I}}}_{{\text{revc}}}\left({{\text{e}}}^{\left(\frac{{{\text{V}}}_{{\text{Pg}}}*{\text{q}}+{{\text{I}}}_{{\text{Pg}}*}{{\text{N}}}_{{\text{s}}}*{{\text{R}}}_{{\text{ss}}}}{\uplambda *{\text{K}}*{\text{T}}*{{\text{N}}}_{{\text{S}}}}\right)}-1\right)$$

11 $${{\text{P}}}_{{\text{Pg}}}={{\text{I}}}_{{\text{Pg}}}*{{\text{V}}}_{{\text{Pg}}}=\left({{\text{I}}}_{{\text{Pg}}}-{{\text{I}}}_{{\text{revc}}}\left({{\text{e}}}^{\left(\frac{{{\text{V}}}_{{\text{Pg}}}*{\text{q}}+{{\text{I}}}_{{\text{Pg}}*}{{\text{N}}}_{{\text{s}}}*{{\text{R}}}_{{\text{ss}}}}{\uplambda *{\text{K}}*{\text{T}}*{{\text{N}}}_{{\text{S}}}}\right)}-1\right)\right){{\text{V}}}_{{\text{Pg}}}$$

12 $${{\text{I}}}_{{\text{LK}}}={{\text{I}}}_{{\text{Pg}}}-{{\text{I}}}_{{\text{revc}}}\left({{\text{e}}}^{\left(\frac{{{\text{V}}}_{{\text{Pg}}}*{\text{q}}+{{\text{I}}}_{{\text{Pg}}*}{{\text{N}}}_{{\text{s}}}*{{\text{R}}}_{{\text{ss}}}}{\uplambda *{\text{K}}*{\text{T}}}\right)}-1\right)-\frac{{{\text{V}}}_{{\text{LK}}}+{{\text{I}}}_{{\text{LK}}}*{{\text{R}}}_{{\text{ss}}}}{{{\text{R}}}_{{\text{st}}}}$$

13 $${{\text{I}}}_{{\text{PV}}}={{\text{I}}}_{{\text{Ph}}}-{{\text{i}}}_{{\text{ore}}}\left({{\text{e}}}^{\frac{{\text{q}}({{\text{V}}}_{{\text{PV}}}+{{\text{I}}}_{{\text{PV}}}{{\text{n}}}_{{\text{s}}}*{{\text{R}}}_{{\text{s}}})}{\upeta *{\text{K}}*{\text{T}}{*{\text{N}}}_{{\text{s}}}}}-1\right)-\frac{{{\text{V}}}_{{\text{PV}}}+{{\text{I}}}_{{\text{PV}}}*{{\text{R}}}_{{\text{se}}}}{{{\text{R}}}_{{\text{su}}}}$$

In the two-diode circuit, there are four junctions are existed to enhance the power supply rating of the sunlight system^22^. Here, the band gap energy plays a major role in transferring the electrons from one direction to another direction. The required variables for the design of the 3-diode circuit sunlight system are I_Pg_, R_St_, R_Ss_, I_Dj_, I_Dk_, ʎ1, plus ʎ2 respectively. The major issue of one diode PV module is junction recombination losses which are limited by using the 2-diode model sunlight system. In the 2-diode PV circuit, there is a leakage in grain boundaries. So, one more diode is included in the two-diode circuit to form the triple-diode sunlight system which is explained in Fig. 4a,b. The overall parameters evaluated in this system are nine which are named as I_Pg_, R_St_, I_DL_, R_Ss_, I_Dj_, I_Dk_, ʎ1, ʎ2, plus ʎ3.

14 $${{\text{I}}}_{{\text{LK}}}={{\text{I}}}_{{\text{Pg}}}-{{\text{I}}}_{{\text{revc}}1}\left({{\text{e}}}^{\left(\frac{{{\text{V}}}_{{\text{Pg}}}*{\text{q}}+{{\text{I}}}_{{\text{Pg}}*}{{\text{N}}}_{{\text{s}}}*{{\text{R}}}_{{\text{ss}}}}{\uplambda 1*{\text{K}}*{\text{T}}}\right)}-1\right)-{{\text{I}}}_{{\text{revc}}2}\left({{\text{e}}}^{\left(\frac{{{\text{V}}}_{{\text{Pg}}}*{\text{q}}+{{\text{I}}}_{{\text{Pg}}*}{{\text{N}}}_{{\text{s}}}*{{\text{R}}}_{{\text{ss}}}}{\uplambda 2*{\text{K}}*{\text{T}}}\right)}-1\right)-\frac{{{\text{V}}}_{{\text{LK}}}+{{\text{I}}}_{{\text{LK}}}*{{\text{R}}}_{{\text{ss}}}}{{{\text{R}}}_{{\text{st}}}}$$

15 $${{\text{I}}}_{{\text{LK}}}={{\text{I}}}_{{\text{Pg}}}-{{\text{I}}}_{{\text{revc}}1}\left({{\text{e}}}^{\left(\frac{{{\text{V}}}_{{\text{Pg}}}*{\text{q}}+{{\text{I}}}_{{\text{Pg}}*}{{\text{N}}}_{{\text{s}}}*{{\text{R}}}_{{\text{ss}}}}{\uplambda 1*{\text{K}}*{\text{T}}}\right)}-1\right)-{{\text{I}}}_{{\text{revc}}2}\left({{\text{e}}}^{\left(\frac{{{\text{V}}}_{{\text{Pg}}}*{\text{q}}+{{\text{I}}}_{{\text{Pg}}*}{{\text{N}}}_{{\text{s}}}*{{\text{R}}}_{{\text{ss}}}}{\uplambda 2*{\text{K}}*{\text{T}}}\right)}-1\right)-{{\text{I}}}_{{\text{S}}}$$

16 $${{\text{I}}}_{{\text{S}}}={{\text{I}}}_{{\text{revc}}3}\left({{\text{e}}}^{\left(\frac{{{\text{V}}}_{{\text{Pg}}}*{\text{q}}+{{\text{I}}}_{{\text{Pg}}*}{{\text{N}}}_{{\text{s}}}*{{\text{R}}}_{{\text{ss}}}}{\uplambda 3*{\text{K}}*{\text{T}}}\right)}-1\right)+\frac{{{\text{V}}}_{{\text{LK}}}+{{\text{I}}}_{{\text{LK}}}*{{\text{R}}}_{{\text{ss}}}}{{{\text{R}}}_{{\text{st}}}}$$

### Process involved in the solar PV cell parameters extraction

**Figure 5:**
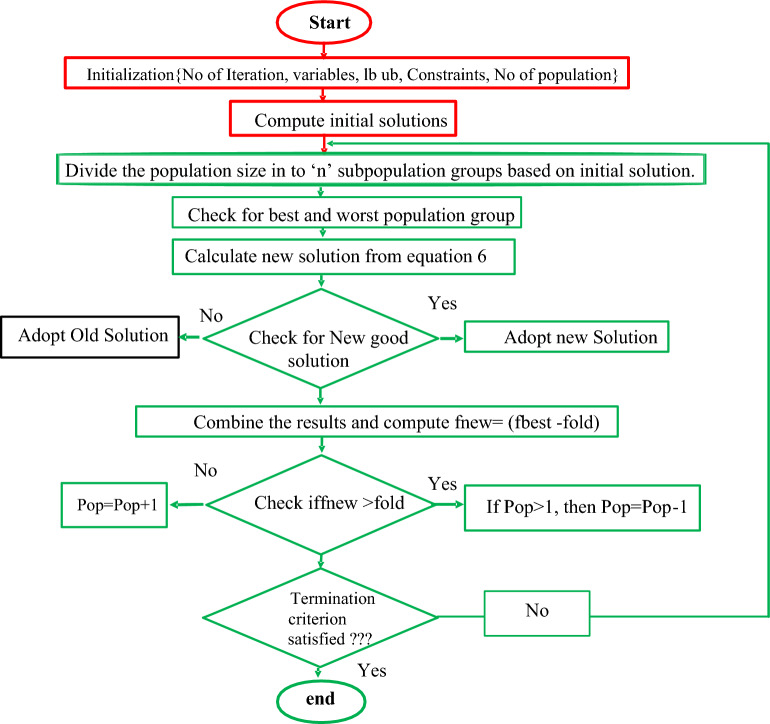
Self-adaptive modified Rao algorithm.

The process involved in the parameters evaluation of the sunlight system are collection of a set of experimental data by utilizing the P–V and I–V curves^23^. Here, the fitness function is defined on the nonlinear characteristics of the sunlight system and it gives either the local maxima or the local minima. The main objective of this article is the identification of error constraints between the evaluated data, plus experimental setup data. The utilized fitness function of this proposed sunlight system is illustrated in Fig. 5. Based on Eq. (17), the RMSE value is determined with the help of actual experimental data and calculated data. The objective function of the proposed system is defined in Eq. (18) which is called a transcendental equation. From the literature study, many researchers represented the issue of the transcendental equation on solving the sunlight parameters extraction. Here, this issue is limited by using the dichotomy method.

17 $${\text{f}}\left({{\text{V}}}_{{\text{LK}}},{{\text{I}}}_{{\text{LK}}},\mathrm{\varnothing }\right)={{\text{I}}}_{{\text{Pg}}}-{{\text{I}}}_{{\text{revc}}1}\left({{\text{e}}}^{\left(\frac{{{\text{V}}}_{{\text{Pg}}}*{\text{q}}+{{\text{I}}}_{{\text{Pg}}*}{{\text{N}}}_{{\text{s}}}*{{\text{R}}}_{{\text{ss}}}}{\uplambda 1*{\text{K}}*{\text{T}}}\right)}-1\right)-{{\text{I}}}_{{\text{revc}}2}\left({{\text{e}}}^{\left(\frac{{{\text{V}}}_{{\text{Pg}}}*{\text{q}}+{{\text{I}}}_{{\text{Pg}}*}{{\text{N}}}_{{\text{s}}}*{{\text{R}}}_{{\text{ss}}}}{\uplambda 2*{\text{K}}*{\text{T}}}\right)}-1\right)- {{\text{I}}}_{{\text{revc}}3}\left({{\text{e}}}^{\left(\frac{{{\text{V}}}_{{\text{Pg}}}*{\text{q}}+{{\text{I}}}_{{\text{Pg}}*}{{\text{N}}}_{{\text{s}}}*{{\text{R}}}_{{\text{ss}}}}{\uplambda 3*{\text{K}}*{\text{T}}}\right)}-1\right)-\frac{{{\text{V}}}_{{\text{LK}}}+{{\text{I}}}_{{\text{LK}}}*{{\text{R}}}_{{\text{ss}}}}{{{\text{R}}}_{{\text{st}}}}-{{\text{I}}}_{{\text{LK}}}$$

18 $$\mathrm{Min }({\text{F}}(\uptheta ))=\sqrt{\frac{1}{{\text{m}}}{{\sum }_{{\text{n}}=1}^{{\text{m}}}\left({{\text{I}}}_{{\text{n}}}-{{\text{I}}}_{{\text{n}},\mathrm{ ext}}(\uptheta )\right)}^{2}}$$

## Proposed technique for parameters extraction

In the dichotomy approach, the number of iterations required for the identification of suitable sunlight parameters is much less, and it needed less convergence time for developing the solar cells. Here, the intermediate theorem is applied to the solar system by selecting a function f(n). The function is working under continuous conditions in the time duration [u, v] then the variables f(u), plus f(v) have opposite signs. As a result, the parameter “w” exists in the middle of the parameters U, plus V. With the help of the above information, the sunlight parameters are obtained by utilizing the metaheuristic technique. In this metaheuristic method, at starting, the unknown parameters are determined and which are applied to the fitness function to check the output of the algorithm. In the second state, the adaptive modified Rao algorithm is selected for extracting the sunlight system values. In the third state, the dichotomy concept is included with the required objective function. In the fourth state, the all-PV cell circuits are used to identify their parameters with unique changes in the proposed algorithm to achieve the optimal RMSE value. The proposed modified Rao algorithm output parameters are compared with other algorithms at benchmark conditions.

The functioning flow of the modified Rao algorithm is illustrated in Fig. 5. From Fig. 5, it is identified that the Rao concept doesn’t involve any data sets for extracting the sunlight system parameters, and it works in a straightforward situation without any uncertainties. In the first iteration of the Rao algorithm, the global and worst solutions are evaluated by applying Eq. (19). In Ref.^24^, the authors used the self-modified Rao concept which works quite similarly to the Rao. In this modified Rao method, all the populations are split into various groups to obtain the different solutions of the PV parameters. Here, all the particles search the entire utilized region to get the best optimal solution. The presently available solutions are cross-verified with the already available solutions to improve the accuracy of the sunlight system.

19 $$ {\text{U}}\__{{{\text{new}}}} = {\text{ U}}\__{{{\text{old}}}} + {\text{ z}}_{{1}} \left( {{\text{U}}_{{{\text{best}}}} {-}{\text{ U}}_{{{\text{worst}}}} } \right) $$

### Ethical approval

This paper does not contain any studies with human participants or animals performed by any of the authors.

## Discussion of results

### 1st case study: 2-diode PV circuit of RTC—France

**Figure 6:**
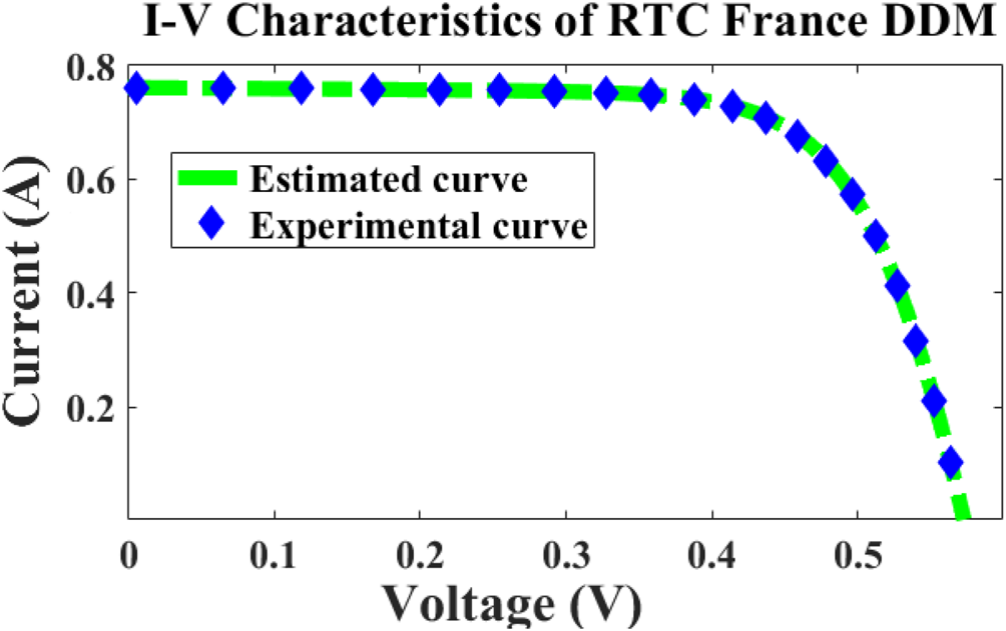
France RTC solar cell I–V curves.

**Figure 7:**
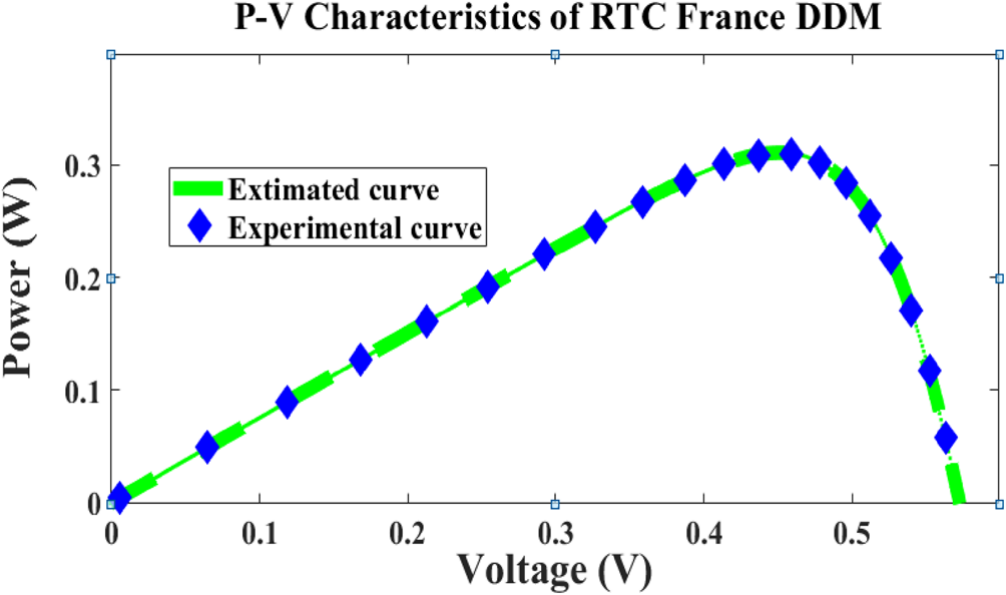
France RTC solar cell P–V curves.

In this case, the 2-diode model, and 3-diode model sunlight systems are selected for extracting the efficiency of the modified Rao method. Here, a 58 mm diameter solar system is selected which is made up of silicon material for commercial applications, and it is an RTC France cell. The overall experimental investigation has been done at 35 °C and 1000 W/m^2^ irradiation value. The RTC France solar cell data sheet is given in Table 1. The available parameters of the sunlight systems by applying the different algorithms are mentioned in Table 2, plus Table 4. The obtained RMSE value for the seven parameters-based 2-diode circuit sunlight system is 7.33167 × 10^–4^. Also, the ten parameters were determined for the sunlight system with an accuracy of 7.33167 × 10^–4^. The determined RMSE values for the solar cells are given in Table 4. The proposed method evaluated theoretical and experimental RTC-France solar PV parameters are indicated in Fig. 6, plus Fig. 7. From Fig. 6, and Fig. 7, it is identified that the RMSE value is much less for both the theoretical and experimental RTC-France Solar Cells.

**Table 1:** Selected RTC France sunlight system datasheet.

Variable	Values	Values
Open circuited voltage V_oc_	0.5728 V	16.778 V
Peak current (Imp)	0.7963A	0.9082A
Peak voltage (Vmp)	0.4440	12.714
Boltzmann constant (K_i_)	0.0350%/C	–
Total cells available (N)	1	36
Short circuited current (I_Sc_)	0.7721A	1.028A

**Table 2:** Extracted parameters of 2-diode sunlight system by applying various metaheuristic methods.

Applied techniques	Parameters							
	I_pg_ (Amp)	I_recvj_ (µA)	I_recvk_ (µA)	ʎ1	ʎ2	R_ss_ (Ω)	R_St_ (Ω)	RMSE × 10^–4^
**MRAODT**	**0.7511**	**2.20789**	**0.07432**	**2.19**	**1.29900**	**0.03765**	**57.897**	7.33167
MPSO^25^	0.76165	0.92	0.08356	1.9117	1.391265	0.038122	56.1856	7.4923
HPCPSO^26^	0.77171	0.006167	0.98234	1.42376	1.910331	0.384194	56.7529	7.891201
AMPSO^27^	0.771867	0.006892	0.19921	1.489123	2.089432	0.038229	57.004367	7.418932
TLA-PSO^28^	0.770781	0.21989	0.723119	1.445012	2.100762	0.037001	56.000124	9.72105
MHNMT^29^	0.771286	0.21246	0.719912	1.461209	2.27899	0.037812	55.983410	9.912672
CSABT^30^	0.77067	0.751131	0.245612	2.14012	1.449991	0.037832	56.002341	9.673216
EJAYA^31^	0.774521	0.228915	0.876612	1.399342	2.29234	0.042671	56.21045	9.9011278
WOBDEA^32^	0.771067	0.220528	0.723451	1.470127	2.941671	0.037899	56.239817	9.9887110

Significant values are in bold.

The total number of iteration counts required for the evaluation of the parameter RTC France sunlight system cell by using MRAODT is less, and its convergence time is also low when associated with the other nature-inspired metaheuristics. Finally, the RMSE value of the modified Rao-based DT controller is low and it takes 100 iterations to achieve the highly accurate solar system parameters. The required iterations, plus the population value summary of the proposed method along with the slider mode optimization are illustrated in Tables 2 and 3. Table 3, it evaluated that the population and iteration values of the optimization algorithm increased then the RMSE value was reduced. Finally, the convergence time of the optimization method is increased then the error value is reduced.

**Table 3:** A detailed comprehensive investigation of the proposed method with sliding mode observer.

Iterations value	MRAODT-RMSE	ASOA-RMSE	Population value	Time
53	1.39101 × 10^–03^	1.41927 × 10^–03^	28	7 s
167	1.073189 × 10^–04^	1.31892 × 10^–04^	44	18 s
261	9.62186 × 10^–04^	9.83178 × 10^–04^	65	29 s
506	**7.41103** × 10^–04^	9.89441 × 10^–04^	106	38 s

Significant values are in bold.

### 2nd case study: 3-diode and 1-diode photo cell watt PWP207

Here, the Photo 3-diode, plus 1-diode Cells are utilized for commercial application and it is indicated as Watt PWP207. The three-diode sunlight system's objective function is to determine the seven parameters and five variables for a 1-diode sunlight system. The utilized cells for both the systems are 36 which are working at 45 °C temperature and 1000 W/m^2^ irradiance. The evaluated proposed method RMSE is equated with the already existing optimization algorithms as shown in Tables 4 and 5.

**Table 4:** Analysis of modified Rao method for 3D RTC solar system.

Techniques	I_Pg_ (Amp)	I_revcj_ (µA)	I_recvk_ (µA)	I_recvl_ (µA)	ʎ_1_	ʎ_2_	ʎ_3_	R_Ss_ (Ω)	R_St_ (Ω)	RMSE × 10^–4^
**MRAODT**	**0.750024**	**1.82771**	**5.876E−6**	**0.007278**	**1.78956**	**1.36915**	**1.3689**	**0.0326192**	**57.6721**	**7.299978**
MGBA^33^	0.76912	0.86139	0.219897	0.0086751	1.9127865	1.4561	1.96712	0.0359813	56.22189	9.7245621
TLBOT^34^	0.770145	0.245612	0.221756	0.456127	1.436741	2.00231	2.00145	0.0361207	55.87452	9.912786
ACSA^35^	0.772231	0.002675	0.167321	0.310076	1.8367345	2.00789	1.46712	0.037119	59.67545	9.9991231
MOWOA^36^	0.771539	0.325632	0.23146	0.461290	1.397812	2.01241	2.005612	0.03711290	56.991254	9.7198987
AMFA^37^	0.778231	0.4399871	0.08147	0.019675	1.781231	1.31279	1.470017	0.036894423	54.11278	10.89967

Significant values are in bold.

**Table 5:** A comprehensive investigation of the proposed method for Photo-Watt PWP-201.

Techniques	Parameters					
	I_Pg_ (A)	I_recv_ (µA)	ʎ	R_Ss_ (Ω)	R_St_ (Ω)	RMSE × 10^–3^
**MRAODT**	**1.01317**	**1.179012**	**44.18921**	**1.501273**	**678.881**	**1.61289**
MPSO^38^	1.021989	2.459891	1.303245	1.2210786	755.108	2.06732
WDA with PSO^39^	1.021897	2.5067812	1.311386	1.2243210	742.5218	2.030892
HCPSA^40^	1.0318921	2.508956	1.326754	1.2310876	742.67342	2.0200134
VAPPSO^41^	1.0308912	2.578034	1.319878	1.2332007	820.17603	2.018976
ASDT^42^	1.0290909	3.4789121	1.330089	1.2102563	980.22310	2.5001245
MHNMT^43^	1.0296712	3.4678198	1.3491207	1.2100183	980.89116	2.4197818

Significant values are in bold.

### 3rd case study: experimental analysis of 1-diode, and 3-diode sunlight systems

**Figure 8:**
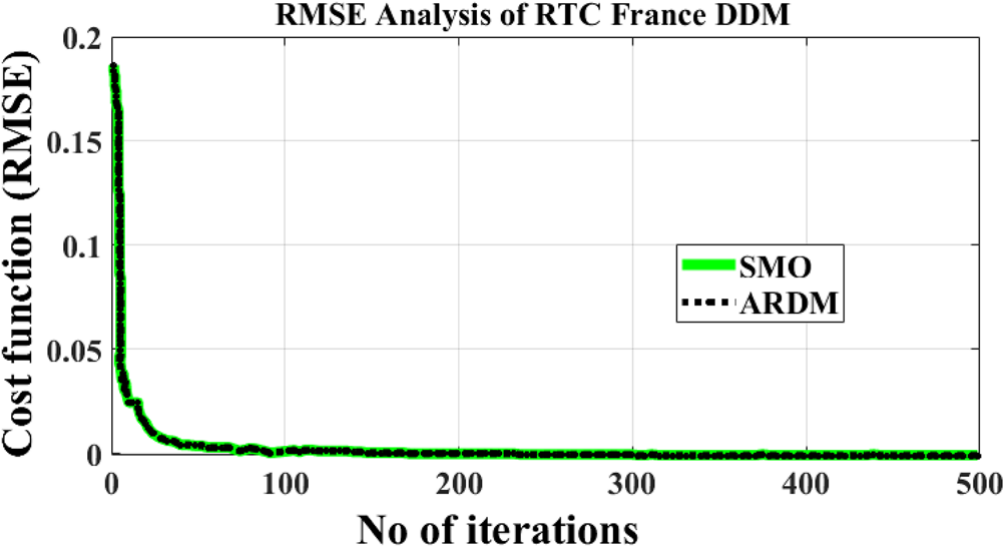
Sunlight system convergence characteristics.

**Figure 9:**
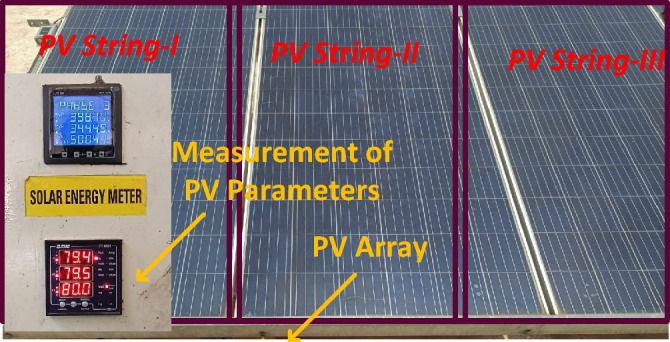
Tested setup of sunlight system.

**Figure 10:**
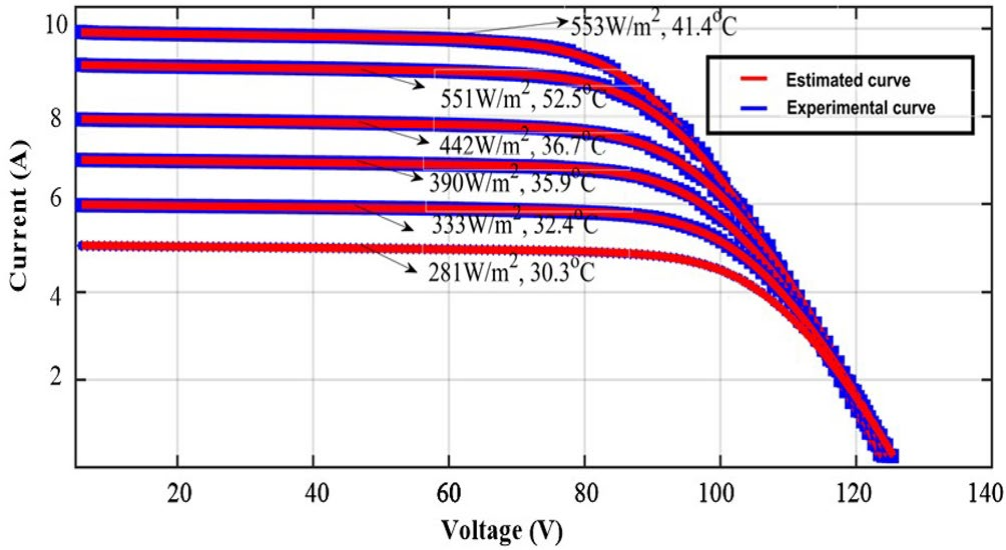
GL100 model PV array nonlinear curves at different temperature values.

The proposed method is investigated by selecting the large-scale system. The evaluated experimental waveforms for the 1-Diode system are explained in Fig. 8. Here, “3” solar strings are utilized, and those strings are consisted of “6” modules. The model GL-M303 monocrystalline PV module along with “36” works as a local consumer application. The model PROVA1011 is used for investigating the nonlinear curves of the sunlight system at multiple sunlight temperatures and irradiation conditions. The data sets provide the electrical characteristics of sunlight systems. In this case, the proposed technique is analysed for a 1-Diode cell for estimating “5” parameters of the sunlight system as given in Table 6. The nonlinear curves of the LM100 model PV array are illustrated in Fig. 9. From Table 6, and Fig. 10, the proposed modified Rao method predicts the sunlight parameters accurately under various environmental conditions. Finally, the 3-diode sunlight system experimental parameters are given in Table 7.

**Table 6:** 2-Diode-based sunlight system real-time performance analysis.

Environmental conditions		Techniques	Evaluated parameters					*RMSE*
Irradiance	Temperature		*I*_*Pg*_ (A)	*Irecv *(µA)	ʎ	R_Ss_ (Ω)	R_St_ (Ω)	
548 W/m^2^	40.21 °C	**MRAODT**	**9.8991**	**0.004871**	**6.12012**	**2.67234**	**361.2198**	**0.0245**
		ABC with TLA	9.9914	0.0012	216.910	2.789247	366.978	0.049897
521 W/m^2^	53.18 °C	**MRAODT**	**9.167843**	**0.0067**	**8.2078**	**2.42310**	**413.08964**	**0.032134**
		ABC with TLA	8.108732	0.01121	7.32456	2.59897	424.89563	0.058921
438 W/m^2^	36.7 °C	**MRAODT**	**8.01287**	**1.197832**	**6.3684**	**2.44431**	**423.23221**	**0.019978**
		ABC with TLA	8.10287	0.08902	221.88	2.7621	420.1764	0.04123
394 W/m^2^	34.89 °C	**MRAODT**	**7.14321**	**0.0023**	**6.09122**	**2.70897**	**488.91564**	**0.03231**
		ABC with TLA	7.9823	0.0056	231.119	2.7178	512.43	0.03989
348 W/m^2^	33.22 °C	**MRAODT**	**6.10098**	**0.0023**	**6.05672**	**2.70271**	**564.2216**	**0.0078**
		ABC with TLA	6.7689	0.0038	228.129	2.59897	581.33	0.0110
294 W/m^2^	31.19 °C	**MRAODT**	**5.100782**	**0.0033**	**6.31892**	**2.72319**	**611.234**	**0.0045**
		ABC with TLA	5.16702	0.0029	217.9023	2.70087	622.768	0.0156

Significant values are in bold.

**Table7:** 3-Diode-based sunlight system real-time performance analysis.

	MRAODT	ASOA^44^	MRAODT	ASOA	MRAODT	ASOA	MRAODT	ASOA	MRAODT	ASOA	MRAODT	ASOA
Variables	G = 562, T = 42.21		G = 568, T = 53.7		G = 451, T = 37.8		G = 392, T = 36.21		G = 333, T = 32.4		G = 281, T = 30.3	
*RMSE*	0.046723	0.0456	0.04989	0.0499	0.0322	0.030989	0.0310	0.030021	0.01799	0.02767	0.014410	0.01144
*ʎ*_*1*_	5.55734	6.11978	8.994521	6.3386	10.0021	229.989	9.1782	224.23	6.19978	223.787	0.88123	227.342
*ʎ*_*2*_	6.78675	0	2.199812	0	6.50112	0	2.7671	0	6.21198	0	4.400234	0
*ʎ*_*3*_	6.782456	0	6.098972	0	6.20908	0	6.30021	0	6.886743	0	0.377676	0
*I*_*pg*_	9.99897	9.00567	8.994510	8.0012	8.12098	8.2897	7.02214	7.1781	6.114320	6.1128	5.071145	4.1110
*R*_*Ss*_	2.54673	2.5121	2.610923	2.6583	2.60021	2.4993	2.5992	2.7564	2.70231	2.73214	2.70012	3.7721
*R*_*St*_	371.0713	377.017	422.98912	427.987	440.89721	420.112	516.8920	514.32	581.3245	581.876	636.9912	623.22
*I*_*recv1*_	0.0021	0.045 × 10^–6^	0	0.02987	9.77 × 10^–7^	0.00878	2.05321	0.0087	3.33 × 10^–10^	0.0065	2.81 × 10^–68^	0.0045
*I*_*recv2*_	6.287 × 10^–9^	0	0	0	0	0	0	0	3.12 × 10^–9^	0	6.22 × 10^–31^	0
*I*_*recv3*_	0.00032	0	9.71 × 10^–9^	0	3.59 × 10^–9^	0	5.66 × 10^–9^	0	0	0	3.55 × 10^–9^	0

Significant values are in bold.

## Conclusion

The solar power supply system efficiency is majorly dependent on its accurate PV module design, and the nonlinear characteristics of the sunlight system. Here, the modified Rao with Dichotomy technique is applied to the 1-diode, 2-diode, plus 3-diode solar cell systems to identify their suitable parameters to supply the peak power to the consumers. The total utilized PV modules in this work are eighteen, and the RMSE value is determined for France-RTC, Watt PWP207. From the above simulation and experimental investigation, the proposed MRAODT algorithm extracts the sunlight system parameters with more accuracy under multiple sun temperatures, plus irradiation conditions. The modified Rao algorithm reaches the convergence speed at 489 iterations. The proposed MRAODT method advantages are fast convergence speed, needed low-level iterations for identifying the suitable PV cell variables, more suitable quick changes of sunlight conditions, plus easy adaptability.

## Data Availability

The data used to support the findings of this study are included in the article.

## Abbreviations

MRAODT: Modified Rao dichotomy technique
RES: Renewable energy source
RMSE: Root mean square error
MPSO: Modified particle swarm optimization
HCSPSO: High convergence speed particle swarm optimization
AMPSO: Adaptive modified particle swarm optimization
CSABT: Cuckoo search algorithm-based biogeography technique
EJA: Enhanced Jaya algorithm
ASOA: Adaptive sequential optimization algorithm
MGBA: Modified gradient-based algorithm
TLBOT: Teaching learning based optimization algorithm
ACSA: Adaptive cuckoo search algorithm
MOWOA: Modified opposition-based whale optimization algorithm
AMFA: Adaptive moth flame algorithm
WDA with PSA: Wind driven algorithm with particle swarm algorithm
HCPSA: High convergence particle swarm algorithm
VAPPSO: Variable accelerating parameters of particle swarm optimization
ASDT: Adaptive successive discretization technique
MHNMT: Modified hybrid Nelder-Mead technique
ABC-TLA: Artificial bee colony teaching learning algorithm
WOBDEA: Whale optimization-based differential evolutionary algorithm
